# Vitamin D and Cardiovascular Diseases: From Physiology to Pathophysiology and Outcomes

**DOI:** 10.3390/biomedicines12040768

**Published:** 2024-03-30

**Authors:** Matteo Nardin, Monica Verdoia, Simone Nardin, Davide Cao, Mauro Chiarito, Elvin Kedhi, Gennaro Galasso, Gianluigi Condorelli, Giuseppe De Luca

**Affiliations:** 1 Department of Biomedical Sciences, Humanitas University, Via Rita Levi Montalcini 4, Pieve Emanuele, 20090 Milan, Italy; 2 Internal Medicine, Department of Medicine, ASST Spedali Civili di Brescia, 25123 Brescia, Italy; 3 Division of Cardiology, Ospedale degli Infermi, ASL Biella, 13875 Biella, Italy; 4 Department of Translational Medicine, Eastern Piedmont University, 28100 Novara, Italy; 5 U.O. Clinica di Oncologia Medica, IRCCS Ospedale Policlinico San Martino, 16132 Genova, Italy; 6 Department of Internal Medicine and Medical Sciences, School of Medicine, University of Genova, 16126 Genova, Italy; 7 Department of Cardiology, Humanitas Gavazzeni Hospital, 24125 Bergamo, Italy; 8 Department of Cardiovascular Medicine, IRCCS-Humanitas Research Hospital, 20089 Rozzano, Italy; 9 McGill University Health Center, Montreal, QC H3G 1A4, Canada; 10 Department of Cardiology and Structural Heart Disease, University of Silesia, 40-032 Katowice, Poland; 11 Department of Medicine, Surgery and Dentistry, University of Salerno, 84081 Baronissi, Italy; 12 Division of Cardiology, AOU “Policlinico G. Martino”, Department of Clinical and Experimental Medicine, University of Messina, 98122 Messina, Italy; 13 Division of Cardiology, IRCCS Hospital Galeazzi-Sant’Ambrogio, 20157 Milan, Italy

**Keywords:** cholecalciferol, vitamin D, cardiovascular disease, pathophysiology, outcome

## Abstract

Vitamin D is rightly recognized as an essential key factor in the regulation of calcium and phosphate homeostasis, affecting primary adequate bone mineralization. In the last decades, a more complex and wider role of vitamin D has been postulated and demonstrated. Cardiovascular diseases have been found to be strongly related to vitamin D levels, especially to its deficiency. Pre-clinical studies have suggested a direct role of vitamin D in the regulation of several pathophysiological pathways, such as endothelial dysfunction and platelet aggregation; moreover, observational data have confirmed the relationship with different conditions, including coronary artery disease, heart failure, and hypertension. Despite the significant evidence available so far, most clinical trials have failed to prove any positive impact of vitamin D supplements on cardiovascular outcomes. This discrepancy indicates the need for further information and knowledge about vitamin D metabolism and its effect on the cardiovascular system, in order to identify those patients who would benefit from vitamin D supplementation.

## 1. Introduction

Cardiovascular diseases continue to be the leading causes of mortality and morbidity worldwide. While significant progress has been made in the effective prevention of communicable diseases, particularly infections, and maternal–fetal mortality [1], this success has resulted in longer average life expectancies, leading to a more pronounced impact of cardiovascular conditions on public health [2].

Despite improvements in the treatment of the acute phase of cardiac diseases, such as myocardial infarction (MI) [3,4,5,6,7,8,9], primarily caused by atherothrombosis in the coronary vessels, the outcomes remain unsatisfactory in high-risk patient subgroups [10,11,12]. Therefore, there is a need for further research to explore new risk factors [13,14,15,16,17] and therapies [18] in coronary artery disease (CAD). A comprehensive understanding of the pathogenetic process is still lacking and, among the investigated factors, vitamin D has been hypothesized to play a role in the atherosclerosis process.

Since the early 20th Century, vitamin D has been shown to prevent and treat bone diseases including rickets and osteomalacia. Beyond its role in calcium and phosphate homeostasis, vitamin D has been significantly associated with cardiovascular disease [19,20]. The key rationale for this relationship is attributed to the transcription role of vitamin D, which has been proved to modulate the expression of approximately 3% of the human genome. Various observational studies have reported a significant association between vitamin D deficiency and various cardiovascular conditions, including but not limited to arterial stiffness, hypertension, atherosclerosis, and left ventricular hypertrophy [21,22]. Mechanisms regulated by vitamin D include endothelial dysfunction, cardiomyocyte function, and the calcification of valves and vessels [21,22,23].

In this review we aim to provide an overview of current evidence regarding the role of vitamin D in the cardiovascular system, covering molecular aspects and different clinical scenarios.

## 2. Methods and Search Strategy

After outlining the structure of our manuscript, we systematically searched the main medical electronic databases, such as PubMed, Medline, Scopus, Cochrane, and Google Scholar, utilizing specific keywords for each section.

For ‘vitamin D’, which was included in every search, we used the following set of terms: vitamin D, Vitamin D3, Vitamin D2, 25-hydroxyergocalciferol, 25-hydroxycholecalciferol, calcifediol, ercalcidiol, 1,25-dihydroxycholecalciferol, calcitriol, 1,25-dihydroxyergocalciferol, ercalcitriol, vitamin D binding protein, and vitamin D receptor. For ‘vitamin D metabolism’, we used terms such as metabolism, anabolism, catabolism, skin, liver, kidney, food, and hydroxylase. For ‘Endothelial dysfunction and inflammation’, we utilized terms such as endothelial dysfunction, inflammation, cytokines, reactive oxygen species, pro-inflammatory, interleukin, tumor necrosis factor, immunity, and immune cells. For ‘coronary artery disease’, we included terms such as coronary artery disease, atherosclerosis, lipid, lipoprotein, calcification, metabolic dysfunction, diabetes, acute coronary syndrome, myocardial infarction, and myocardial ischemia. For ‘platelet aggregation’, terms such as platelets, aggregation, aggregometry, and platelet reactivity were used. For ‘systemic arterial hypertension’, we utilized terms including hypertension, renin–angiotensin–aldosterone system, blood pressure, vascular resistance, reactive oxygen species, incidence, and prevalence. For ‘aortic stenosis’, we employed terms such as aortic valve stenosis, degenerative aortic stenosis, and valve calcification. For ‘heart failure’, terms such as heart failure, dyspnea, congestion, preserved ejection fraction, reduced ejection fraction, and left ventricle were used. For ‘genetics’, terms such as single nucleotide polymorphism, genetics, allele variant, and haplotype were utilized. For ‘therapeutic considerations’, terms such as trial, randomized clinical trial, supplement, outcome, mortality, and cardiovascular events were included. Priority was given to papers published since January 2004 and those found in indexed peer-reviewed journals. Each section was organized to present pre-clinical evidence followed by clinical findings. The therapeutic and outcome aspects were collectively addressed in a dedicated section.

## 3. Vitamin D Metabolism

**Figure 1:**
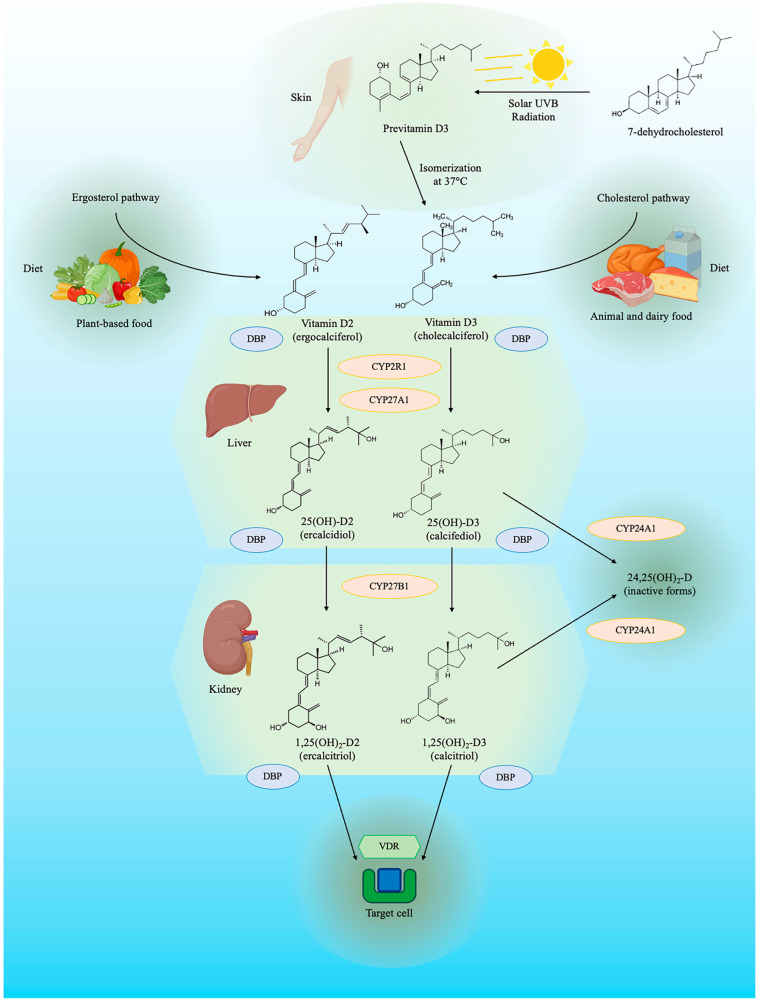
**Vitamin D synthesis pathway.** The figure depicts the synthesis of vitamin D and its metabolites derived by ergosterol and cholesterol. CYP24A1 = cytochrome P450 family 24 subfamily A member 1, also named 25(OH)-D 24-hydroxylase; CYP27A1 = cytochrome P450 family 27 subfamily A member 1, also named sterol 27-hydroxylase; CYP27B1 = cytochrome P450 family 27 subfamily B member 1, also named 25(OH)-D 1α-hydroxylase; CYP2R1 = cytochrome P450 family 2 subfamily R member 1, also named vitamin D 25-hydroxylase; DBP = vitamin D binding protein; UVB = ultraviolet B; VDR = vitamin D receptor.

The metabolism of vitamin D comprises intricate pathways (Figure 1) involving the skin, liver, kidney, and solar light to obtain the final active compound. It is important to note that the term vitamin D refers to a group of secosteroids derived from dietary intake or human cell production. In animal cells, the initial step involves 7-dehydrocholesterol, a precursor of cholesterol. Human skin cells also produce this compound, acting as pro-vitamin D. After exposure to solar ultraviolet B beams, the B ring of the steroid nucleus breaks, resulting in a secosteroid, which is called pre-vitamin D. This pre-vitamin D is then converted through spontaneous isomerization by a thermal reaction into cholecalciferol, also known as vitamin D3. The skin’s production of vitamin D3 is proportional to sun exposure and the quantity of melatonin. Vitamin D3 levels can also be increased through dietary intake from animal-based foods, mainly dairy products. Ingestion of plant-based food leads to the absorption of a similar compound named vitamin D2, derived from the plant cell steroid ergosterol. Both vitamin D3 and vitamin D2 are metabolized by hepatic 25-hydroxylases, encoded by the gene for cytochrome P450 2R1 (CYP2R1), into 25-hydroxycholecalciferol (25(OH)-D3), also known as calcifediol if from vitamin D3, or into 25-hydroxyergocalciferol (25(OH)-D2), also named ercalcidiol, if from vitamin D2. Routine blood measurements do not differentiate between 25(OH)-D3 and 25(OH)-D2, grouping them both as 25(OH)-D. General recommendations for vitamin D supplements assign the same conversion value to vitamin D3 and D2 [24], due to their similar [25], although not identical [26], ability to elevate serum 25(OH)-D levels. Most steps in the metabolism and actions of vitamins D2 and D3 are overlapping, but evidence suggests that cholecalciferol increases serum 25(OH)-D levels to a greater extent and maintains these higher levels for longer than ergocalciferol does [27], even though both forms are well absorbed in the gut [28,29]. Analogous evidence has been traditionally described in relation to rickets [30,31], but recent data from human transcriptome analyses has shown that gene expression related to immunity differs between vitamin D2 and D3 [32].

The final step involves hydroxylation on carbon 1, mediated by renal 1α-hydroxylase, encoded by gene CYP27B1, leading to the active vitamin D compounds 1,25-dihydroxycholecalciferol (1,25(OH)_2_-D3), also known as calcitriol if from vitamin D3, or 1,25-dihydroxyergocalciferol (1,25(OH)_2_-D2), also named ercalcitriol if from vitamin D2. Biological effects of compounds from vitamin D3 and vitamin D2 are widely accepted as equal, allowing the use of the comprehensive term 1,25(OH)_2_-D for both [33]. Both 25(OH)-D and 1,25(OH)_2_-D can by inactivated by 24-hydroylase, producing calcitroic acid (24,25(OH)_2_-D).

Due to the highly lipophilic properties of all vitamin D compounds, they bind to vitamin D binding protein (DBP) and, to a lesser extent, albumin in the bloodstream. Only a limited fraction of vitamin D is unbound to circulating proteins and is considered biologically active. The half-life of 25(OH)-D is significantly higher than that of the active compound 1,25(OH)_2_-D, at 10–20 d vs. 10–20 h. The target of 1,25(OH)_2_-D is the vitamin D receptor (VDR), located in the cytosol of in the nucleus of target cells. After crossing the cell membrane, calcitriol binds to VDR, causing a conformational change in the receptor and leading to heterodimerization with retinoic acid’s X-receptor. This complex then translocates into the nucleus, binding to vitamin D response elements on DNA and enhancing the calcium-binding protein synthesis. Non-genomic effects of vitamin D have been described, mostly involving the release of ionized calcium from intracellular stores.

Recent findings have shown that vitamin D can be activated in extra-renal tissues expressing the 1α-hydroxylase enzyme, suggesting a role of circulating 25(OH)-D as a substrate for local 1,25(OH)_2_-D synthesis. The extra-renal produced 1,25(OH)_2_-D may exert autocrine and paracrine effects. Therefore, the final biological effect of vitamin D is a sum of circulating calcitriol, derived by the kidney, and a locally produced fraction.

Classically, the metabolic pathways are finely regulated by calcium, phosphate, parathyroid hormone (PTH), and fibroblast growth factor 23 (FGF23). The peptide hormone PTH focuses on increasing blood calcium levels by acting on osteoclasts in bone, on calcium reabsorption in renal tubules, and on production of 1,25(OH)_2_-D in the kidney. Negative feedback is mediated by the increase in calcium and calcitriol levels [34,35].

**Figure 2:**
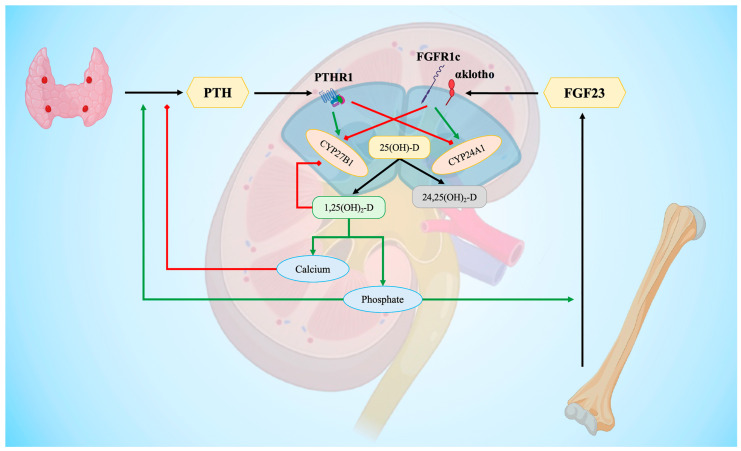
**Hormonal regulation of vitamin D.** The figure shows the hormonal regulation of the final step of vitamin D synthesis and the interplay at renal tubular cells (blue cells) of different signals. Green arrows indicate positive modulation; red arrows indicate inhibition. CYP24A1 = cytochrome P450 family 24 subfamily A member 1, also named 25(OH)-D 24-hydroxylase; CYP27B1 = cytochrome P450 family 27 subfamily B member 1, also named 25(OH)-D 1α-hydroxylase; FGF23 = fibroblast growth factor 23; FGFR1c = fibroblast growth factor receptor 1c; PTH = parathyroid hormone; PTHR1 = parathyroid hormone 1 receptor. Created with BioRender.com.

FGF23 is a compound directly involved in inherited renal phosphate–wasting disease [36]. Released by osteoblasts and osteocytes, its synthesis is stimulated by 1,25(OH)_2_-D [37,38]. In the last decades, it has been identified as a significant regulator of vitamin D metabolism, inhibiting the kidney1α-hydroxylase [39]. Several pre-clinical studies have confirmed the link between bone and kidney mediated by FGF23 [38,40,41], forming a bone–kidney axis in the regulation of active vitamin D (Figure 2).

Emerging evidence in recent years has highlighted the endocrine role of vitamin D, not limited to calcium homeostasis but also affecting a wide range of cardiovascular diseases and components of metabolic syndromes. A key element is the presence of VDR in organs beyond those directly involved in calcium and phosphate regulation. Walters et al. identified VDR in rat hearts in 1986 [42], and VDR has also been found in endothelial cells, vascular smooth muscle cells, cardiac fibroblasts, and platelet surface [43,44,45,46], attributing a broad cardiovascular impact to vitamin D.

## 4. Endothelial Dysfunction and Inflammation

The definition of endothelial dysfunction encompasses a multitude of factors, signals, and compounds that share the common feature of impairing vascular endothelium homeostasis. Various pathological conditions, including atherosclerosis, hypertension, and platelet aggregation, are exacerbated by endothelial dysfunction.

Vitamin D can protect the endothelial function from different types of damage primarily mediated by reactive oxygen species (ROS) [47]. In the leptin-induced endothelial dysfunction model, calcitriol exhibits a protective role on human umbilical vein endothelial cells. This effect is mediated by downregulating vascular inflammatory mediators, such as monocyte chemoattractant protein 1 (MCP-1) and vascular cell adhesion molecule 1 (VCAM-1), along with pro-inflammatory factors, including nuclear factor-kB (NF-kB) [48,49]. A cross-sectional study in obese children observed an inverse association between 25(OH)-D and C-reactive protein (CRP), interleukin (IL)-6, malondialdehyde (MDA), and superoxide dismutase (SOD) [50]. In a randomized control trial with 114 type 2 diabetic patients, vitamin D supplementation led to improvements in HbA1c levels and a reduction in advanced oxidation protein products [51]. Conversely, vitamin D deficiency was found to be significantly associated with endothelial dysfunction [52]. Two meta-analyses reported that vitamin D supplementation significantly reduced CRP, MDA, and tumor necrosis factor-α (TNF-α) [53,54].

In addition to affecting inflammatory mediators, vitamin D could also influence immune cells and their polarization towards a pro-inflammatory phenotype [55]. Previous studies reported a reduction in lymphocytes and inflammatory cytokines secretion after vitamin D administration [56,57]. These findings were consistent among both diabetics and non-diabetics, suggesting the robustness of the association between vitamin D and immunity, irrespective of concomitant comorbidity [58]. Specifically, vitamin D reduced the expression of IL-1β, IL-6 and TNF-α, favoring IL-10 secretion [59,60], and promoted M2 macrophage differentiation while hampering the M1 type [61]. Vitamin D was reported to play a role in reducing neutrophil extracellular traps (NETs), that are deeply involved in the atherothrombotic process [62,63]. Calcitriol inhibits the generation of lymphocyte T helper 1 (TH1) and T helper 17 (TH17) [64,65], while promoting lymphocyte T helper 2 (TH2) and T regulatory (Treg) cells [66,67]. Therefore, a consensus has been reached regarding the essential role of vitamin D in regulating the immune system, marked by a general anti-inflammatory action [68].

## 5. Coronary Artery Disease

Atherosclerosis, closely linked with inflammation, affects both the initiation and progression of coronary artery disease (CAD). The protective role of vitamin D against CAD involves the modulation of the inflammatory response by reducing the expression of IL-1, IL-6, and TNF-α [19,69]. This subsequently decreases CRP and other pro-inflammatory cytokines, limiting the formation of macrophage-derived foam cells, a pivotal element in atherosclerosis progression [70]. Beyond its anti-inflammatory effects, calcitriol is hypothesized to impact lipid homeostasis, particularly by reducing low-density lipoprotein (LDL) uptake by macrophages through the limitation of scavenger receptor expression on the macrophage surface [71]. These findings were confirmed in subjects with various cardiovascular risk factors, including diabetes mellitus and obesity [72].

In clinical studies, serum vitamin D levels were found to be inversely associated with the extent of vascular calcifications [73] and with carotid intima–media thickness [74], which is considered an early marker of atherosclerosis. The concept of a bone–vascular axis has been explored in terms of rationale and evidence, but further clarification is needed to assess its clinical effect [75].

Considering the shared inflammatory signaling pathways between vitamin D and other cardiovascular risk factors, potential confounding interplays may exist. However, a large prospective study, the Health Professionals Follow-up Study, reported that the association between low vitamin D levels and an increased risk of acute myocardial infarction is substantially maintained, irrespective of major cardiovascular risk factors. This study, that included 18,225 men with up to a 10-year follow-up period, showed that patients with vitamin D levels ≥ 30 ng/mL had almost half the adjusted risk of myocardial infarction [76]. Subsequent meta-analyses confirmed a 1.5 times adjusted relative risk of major adverse cardiovascular events for lower (≤60 nmol/L) vs. higher (>60 nmol/L) vitamin D levels [77].

Among patients undergoing percutaneous coronary intervention (PCI), a consistent prevalence of vitamin D deficiency was detected, with a significant relationship with the prevalence and extent of CAD [78]. Similar results were found in patients with diabetes mellitus, although diabetes was not identified as a predictor of vitamin D deficiency [79].

In a subset of patients referred for a ST-segment elevation myocardial infarction (STEMI), vitamin D levels were inversely related to total cholesterol and LDL values, regardless of ongoing statin therapy at admission [80], as well as inflammatory biomarkers [81].

In the extensive Multi-Ethnic Study of Atherosclerosis (MESA), which involved 6436 patients with CAD and an observational period exceeding 8 y, individuals of the white race with lower serum 25(OH)-D concentration (<20 ng/mL) exhibited an elevated risk of experiencing cardiovascular events [82]. Also, among patients admitted for an acute coronary syndrome, several studies have confirmed a higher incidence of major adverse cardiovascular events [83,84,85], including impaired reperfusion in STEMI patients [86].

However, no significant link has been found between severe vitamin D deficiency (<10.2 ng/mL) and peri-procedural MI among patients undergoing PCI [87]. The main reason for this could be attributed to the different pathogenesis of acute and peri-procedural MI, with the latter strongly influenced by procedural features, including the instruments used and pharmacological treatments, such as glycoprotein IIB/IIIA inhibitors [88].

Possible explanations for these results may involve detrimental left ventricular remodeling; Padoan et al. suggested that adverse remodeling after a MI was observed in patients with lower vitamin D levels at baseline (12.6 vs. 18.7 ng/mL), with an increased risk of mortality at follow-up [89]. Definite answers, however, are still lacking, requiring further clarification of underlying mechanisms to design appropriate trials investigating effective therapeutic strategies.

## 6. Platelet Aggregation

Koyama et al. have suggested a pivotal antithrombotic role of vitamin D, reporting that calcitriol regulates thrombomodulin and tissue factor expression, thereby reducing platelet aggregation and thrombogenicity [90]. Furthermore, VDR has been identified in platelet mitochondria [46], indicating a direct role of vitamin D in regulating platelet function [91]. Non-genomic action in platelets is initiated by the binding of vitamin D and VDR, involving the regulation of intracellular calcium concentration, a crucial crossroad for platelet signaling leading to their aggregation [63].

Of particular interest is the impact of vitamin D levels during antiplatelet therapy, which is crucial in CAD patients requiring treatment. In patients on dual antiplatelet therapy (DAPT) with clopidogrel or ticagrelor, lower (≤9.1 ng/mL) vitamin D levels were related to a higher prevalence of high on-treatment residual platelet reactivity (HRPR), although these results did not achieve statistical significance at multivariate analysis [92]. However, in diabetic patients, the inverse relationship finding between HRPR prevalence and vitamin D levels reached statistical significance, especially for new P2Y_12_ inhibitors [93], suggesting a close connection between glycemic and vitamin D pathways in the cardiovascular field [94].

Further confirmations have been provided by Sultan et al., who found a significantly enhanced platelet reactivity among diabetic patients inversely correlated with vitamin D levels. Moreover, calcitriol administration to ex vivo platelets led to a significant reduction in platelet reactivity induced by collagen and ADP [95]. Involvement in the metabolism of homocysteine, a pro-thrombotic substance, has also been suggested for vitamin D [96]. These findings underscore the contribution of vitamin D to regulating platelet activity and thrombus formation. However, the exact molecular pathway induced by non-genomic VDR in platelets needs clarification, as well as the potential contribution of PTH [97].

## 7. Systemic Arterial Hypertension

As the foremost cardiovascular risk factor, systemic arterial hypertension deserves consideration regarding its potential link with vitamin D. The role of vitamin D in endothelial dysfunction, reactive oxygen species (ROS) production, and immune cell regulation could contribute to the development and maintenance of hypertension.

Specific research attention has been dedicated to the renin–angiotensin–aldosterone system (RAAS). In murine models, mice knockout for VDR exhibited higher blood pressure and developed cardiac hypertrophy due to enhanced renin expression, subsequently activating the signaling cascade [98]. The protective role of RAAS inhibitors was found to be more pronounced among human patients with lower values of vitamin D (12.7 ng/mL) [99].

However, initial animal models showed potential interference mediated by secondary hyperparathyroidism, leading to the introduction of a diet enriched with calcium and phosphate to normalize calcium homeostasis and renin concentration [100].

Conversely, aortic tissue in mice subjected to knockout for VDR and on an enriched diet exhibited an attenuated content of elastin and collagen. Additionally, endothelial nitric oxide synthase (eNOS) was reduced by half [100,101]. A complementary investigation in rats confirmed improved vascular tone with chronic calcitriol administration, resulting in upregulation of eNOS, reduction of ROS, and cyclooxygenase-1 (COX-1) expression [102].

Large-scale observational studies have linked vitamin D status to blood pressure values and the incidence of hypertension. In the National Health and Nutrition Examination Survey (NHANES) III, a significant inverse relationship between vitamin D and blood pressure values was reported, irrespective of age, sex, ethnicity, and physical activity [103]. Nevertheless, a noticeable attenuation of this correlation was found after accounting for body mass index and PTH levels, suggesting that PTH might mediate the observed impact of vitamin D on blood pressure [104], potentially as reported for HRPR prevalence in CAD patients [97].

Further investigations have suggested an attenuated age-related increment of blood pressure values, particularly systolic values, in people with normal vitamin D levels [105,106]. A meta-analysis of prospective observational studies reported a lower risk of hypertension of 12% for every 10 ng/mL increment in circulating 25(OH)-D levels [107]. However, other authors have not found the same trend [108], and suggested a more prominent role of PTH in the regulation of blood pressure rather than vitamin D [109], leading to an indeterminate answer to the question related to hypertension.

## 8. Aortic Stenosis

Degenerative aortic valve stenosis has garnered significant research attention in recent decades, particularly due to impressive therapeutic advancements using transcatheter approaches. However, its pathogenesis remains elusive, hindering a comprehensive understanding and the development of preventive strategies to alleviate the condition. Despite sharing several risk factors with CAD, a consistent percentage of patients do not exhibit concomitant significant CAD [110].

The role of vitamin D in degenerative aortic valve stenosis is controversial. It may either promote calcium deposition, leading to an increase in serum calcium, or mitigate the inflammatory environment that could advance valve disease progression. Despite pre-clinical evidence suggesting potential vessel calcification with exposure to high concentrations of vitamin D [111], murine models have shown that vitamin D supplementation does not exacerbate calcium deposition and aortic stenosis grade [112]. A more prominent role has been suggested for PTH. In an in vitro study, valvular endothelial cells exposed to high levels of PTH were found to undergo changes in transcription and protein secretion. This modification also impacts paracrine mediators that interact with valvular interstitial cells, stimulating them to switch their phenotype to an osteogenic profile [113].

Findings from a small-scale study proposed a potential linear relationship between low levels of vitamin D and aortic valve peak flow velocity, as well as stenosis progression [114]. A retrospective study demonstrated significantly lower survival and a higher incidence of aortic valve replacement among patients receiving calcium supplements, with or without vitamin D, compared to those not receiving calcium [115]. Conversely, another study found phosphate, but not calcium or vitamin D, to be significantly related to aortic valve stenosis incidence [116]. A large prospective observational Chinese study suggested a potential protective role of vitamin D in relation to degenerative aortic valve stenosis [117].

Further studies are needed to clarify the mechanisms related to the calcification process involving both valvular endothelial and valvular interstitial cells. The complex interplay of signals is not completely understood [118], making it challenging to definitively state the potential beneficial or detrimental impact of vitamin D.

## 9. Heart Failure

**Figure 3:**
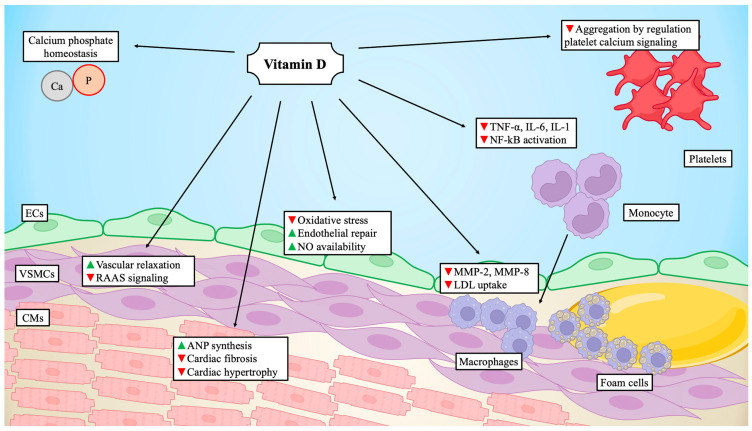
**Vitamin D targets in the cardiovascular field.** The figure shows main targets and effect of vitamin D related to cardiovascular diseases. Green arrowheads indicate increase, red arrowheads indicate inhibition. ANP = atrial natriuretic peptide; Ca = calcium; CMs = cardiomyocytes; ECs = endothelial cells; IL-1 = interleukin-1; IL-6 = interleukin-6; LDL = low-density lipoprotein; MMP-2 = matrix metalloprotease-2; MMP-8 = matrix metalloprotease-8; NF-kB = nuclear factor-kB; NO = nitric oxide; P = phosphate; RAAS = renin angiotensin aldosterone system; TNF-α = tumor necrosis factor-α; VSMCs = vascular smooth muscle cells. Created with BioRender.com.

The term heart failure (HF) denotes a complex syndrome arising from structural and/or functional abnormalities of the heart, resulting in elevated intracardiac pressure and/or inadequate cardiac output at rest and/or during exercise. The pathophysiology of HF is characterized by several mechanisms, including hemodynamic impairment, neurohormonal activation, enhanced inflammation, and cardiac remodeling [119] (Figure 3).

Among the structural alterations observed in HF is left ventricular hypertrophy, typically occurring in response to volume or pressure overload, as seen in conditions such as hypertension or aortic valve stenosis. The thickening of the wall is often accompanied by an increase in chamber dimensions. Concurrently, with the enlargement of cardiomyocytes, interstitial fibrosis occurs, primarily reactive, while fibrosis developing after ischemia and cell loss is termed reparative. In both cases, there is an increase in extracellular matrix components in the myocardium, leading to stiffening of the walls and functional impairment [120].

There is consistent evidence supporting the anti-fibrotic and anti-hypertrophic action of vitamin D. Multipotent mesenchymal cells induced in a fibrotic phenotype, after exposure to 1,25(OH)_2_-D, exhibit decreased production of collagen, transforming growth factor-β1, and an increase in the production of metalloprotease-8 [121]. Moreover, in an animal model with mice subjected to knockout for the VDR gene, Chen et al. demonstrated a direct anti-hypertrophic activity in the heart from vitamin D-VDR activation which, in turn, involves the calcineurin/nuclear factor of activated T cell/modulatory calcineurin inhibitory protein 1 pathway [122].

Observational data revealed that patients with moderate or severe vitamin D deficiency (<20 ng/mL) displayed higher left ventricular wall thickness and diameter than non-deficient patients [123]. This association was also maintained in the context of severe aortic stenosis, demonstrating a linear relationship between vitamin D levels and wall thickness [124]. Similarly, left ventricular mass and myocardial performance index were impaired in a context of low vitamin D levels (<20 ng/mL) in a subset of hypertensive patients with preserved left ventricular ejection fraction [125].

Clinical studies have d a significant relationship between HF and vitamin D deficiency and its impact on outcomes. In a general healthcare population of more than 40,000 patients, vitamin D conferred an adjusted hazard ratio for new-onset HF of 1.31 and 2.01 for low (16–30 ng/mL) and very low (≤15 ng/mL) levels of vitamin D, respectively, compared with the normal value [126], and the general prevalence of HF progressively increased from normal (>30 ng/mL) to very low vitamin D levels. Functional capacity was also impaired, resulting in a consequent increase in hospitalization among patients with HF and vitamin D deficiency (≤10.9 ng/mL) [127].

Results from the NHANES cohort in the early 2000s, including 8351 patients, showed that 89% of patients with HF and CAD were deficient in vitamin D (<30 ng/mL) [128]. Additionally, Liu et al. demonstrated an adjusted hazard ratio of 1.09 (95% CI = 1.00–1.16) (*p* = 0.040) for all-cause mortality or re-hospitalization for HF at 18 months per 10 mmol/L decrease in vitamin D [129].

## 10. Genetics

The primary investigations related to genetic variants in vitamin D pathways have involved DBP, VDR, and hydroxylase enzymes [130]. In relation to DBP, two main single nucleotide polymorphisms (SNPs), rs7041 and rs4588, located in exon 11 of the coding gene, have been studied. In the ARIC study, carriers of the rs7041 substitution, genetically predisposed to low (17.2 ng/mL) 25(OH)-D levels, showed a mild increased risk of stroke at a median follow-up of 20 years [131]. However, no link with ischemic heart disease was found [132]. On the other hand, Powe et al. showed that the T allele of rs7041 was associated with lower levels of DBP, potentially increasing the free quotient of 25(OH)-D [133]; however, the impact on CAD remains undetermined [134]. The rs7041 was also investigated in relation to platelet aggregation: carriers with the G allele, which provides a higher concentration and affinity for vitamin D of DBP, were found to more frequently have HRPR in the case of lower serum vitamin D levels. Moreover, the results of the platelet aggregation ADP-test were significantly inversely related to vitamin D levels in G allele carriers [135].

The main SNPs of the VDR gene investigated affected both exons and intronic regions. The rs731236, also known as the associated restriction enzyme TaqI, is a synonymous T>C substitution on exon 9. A link with cardiovascular disease has been described in Pakistani [136] and Asian populations [137]. In French type 2 diabetics, TaqI, together with two SNPs of intronic regions, rs1544410 (G>A on intron 8) and rs7975232 (A>C on intron 8), was found significantly in linkage disequilibrium and significantly related to the prevalence of CAD [138]. The intronic SNPs rs1544410 (G>A) and rs7975232 (A>C), also named BsmI and ApaI, respectively, from the associated restriction enzyme, have been significantly related to low HDL cholesterol and low vitamin D. Homozygotes for the mutated A allele of BsmI resulted in an increased risk of CAD [139]. In other populations, BsmI but not TaqI significantly correlates with the incidence of CAD [140]. Other authors found no relationship between ApaI or TaqI and CAD, while a significant association was reported for another SNP, rs2228570, also known as FokI (A>T,C,G on exon 2) [141]. This leads to a shorter and more active VDR as homozygotes for the A allele of FokI SNPs had a significantly higher risk of CAD compared to other genotypes [142]. In a retrospective case–control study involving a Caucasian population, only SNP rs2228570 was significantly associated with the development of CV disease, while no influence was observed with the VDR polymorphisms BsmI, TaqI or ApaI [143]. Considering different ethnicities, in a Chinese study, two other SNPs in the non-coding region of the VDR gene, rs2189480 (C>A on the intronic region) and rs3847987 (C>A on the 3′- untranslated region), were found to be significantly related to cardiovascular disease [144]. Other researchers have investigated polymorphisms of VDR in relation to the development of restenosis after a PCI in a large cohort of patients. The main findings were consistent with a lower rate of coronary restenosis after PCI in patients presenting the AA haplotype in block 2 of the VDR gene compared to those with the GG haplotype. Considering that the block 2-AA haplotype has previously been shown to result in increased transcriptional activity of the VDR promoter, it appears that the higher the number of VDR, the lower risk of restenosis [145]. Genetic variants of VDR have been found to be significantly related to degenerative aortic stenosis in a pivotal study investigating the association of vitamin D with valvular disease [146].

Large studies adopting Mendelian randomization analyses investigated specific SNPs in relation to the risk of cardiovascular disease outcomes, including cardiovascular mortality, CAD, MI, and hypertension. Brøndum-Jacobsen et al. [147] and Afzal et al. [148] investigated four SNPs, two related to the 7-dehydrocholesterol reductase (DCHR7) gene (rs7944926 and rs11234027), and two in the CYP2R1 gene (rs10741657 and rs12794714). The main findings showed that genetically determined low 25(OH)-D levels were associated with increased all-cause mortality and cancer mortality, but not with increased cardiovascular mortality. Similarly, no evidence was found in relation to the risk of ischemic heart disease or MI. After selection through genome-wide association, four SNPs related to DCHR7, hydroxylase enzymes, and transport were tested in a Canadian population. The authors’ conclusion was that genetically lowered 25(OH)-D levels were not associated with an increased risk of CAD, suggesting that observational evidence of the relationship between circulating 25(OH)-D levels and CAD was possibly confounded or potentially due to reverse causation [149]. The most recent analyses included a large UK population and used non-linear Mendelian randomization after the identification of 35 genome-wide significant variants. The findings provided genetic evidence for an L-shaped association between 25(OH)-D and cardiovascular risk, which was largely restricted to individuals with low vitamin D status. The amelioration of vitamin D status among individuals with the lowest concentrations (<50 nmol/L) provided the strongest benefit [130].

Results on DNA are not conclusive, despite some evidence, but there are many confounds, and the final effect includes other elements. Studies involving non-coding RNA, in particular microRNA, are ongoing, but the evidence available so far is scant and limited in size in relation to the cardiovascular impact of vitamin D [150].

## 11. Therapeutic Considerations

There is a lack of agreement on optimal circulating vitamin D levels and thresholds to define its deficiency in the general population (Table 1). Moreover, the various types of supplements used, including vitamin D3, vitamin D2, and calcifediol, pose challenges in formulating conclusions.

In relation to lipid parameters, there is consistent heterogeneity in randomized trials, both in terms of baseline vitamin D levels and daily supplementation doses. A meta-analysis of 38 randomized clinical trials demonstrated a small but significant effect of vitamin D compared to a placebo in reducing total cholesterol, LDL, and triglycerides, and increasing high-density lipoprotein (HDL) [151]. Similar results for total cholesterol, LDL, and triglycerides were found in a more recent meta-analysis by Dibaba, including 41 randomized controlled trials with 3434 patients. However, HDL levels were not significantly modified by vitamin D supplements compared to a placebo [152]. Notably, there is evidence of a beneficial interaction between statins and vitamin D levels. The introduction of high-intensity statins in patients undergoing PCI increases vitamin D levels independently of ongoing supplementation and reduces platelet reactivity [153].

Despite robust evidence of the relationship between vitamin D and coronary artery disease (CAD), controversies persist regarding the impact of oral supplementation on cardiovascular outcomes [154]. The main evidence of benefits is based on the improvement of the inflammatory environment, including CRP and IL-6 [81]. Ergocalciferol administration among CAD patients did not result in beneficial changes in vascular function parameters after 3 mo [155]. In a small parallel-group, placebo-controlled, double-blind randomized trial in patients with a prior myocardial infarction (MI), a high dose of cholecalciferol showed no effect on cardiovascular outcomes, including blood pressure or cholesterol levels [156]. Conversely, Chinese CAD patients receiving calcitriol supplementation showed improvement in their SYNTAX score after 6 months [157].

Findings on hypertension are inconclusive. While some studies suggest that vitamin D supplementation, even at high doses, improved blood pressure values in the short term [158], a meta-analysis of 16 clinical trials did not show a significant impact on blood pressure from vitamin D supplements, except for patients with existing cardiometabolic impairment [159]. In young people with vitamin D deficiency, the impact of specific supplements is negligible [160].

The Randomised Evaluation of Calcium Or vitamin D (RECORD) trial, involving 5262 older people randomized to receive cholecalciferol (800 IU/day), calcium (1000 mg/day), cholecalciferol plus calcium, or a placebo, demonstrated a significant reduction in heart failure (HF) events with vitamin D supplementation compared to no supplementation, but no difference in myocardial infarction (MI) or stroke [161]. The VitamIN D treating patients with Chronic heArT failurE (VINDICATE) study, a randomized double-blind controlled trial in elderly patients with HF and vitamin D deficiency, showed a significant improvement in left ventricular structure and function with daily 100 μg (4000 IU) cholecalciferol [162]. However, when considering hard endpoints such as mortality, vitamin D supplementation among deficient patients with HF showed no difference compared to a placebo [163].

The following three large randomized controlled clinical trials investigated effects of vitamin supplementation on cardiovascular disease outcomes as a primary endpoint: the Vitamin D Assessment (ViDA) trial [164], the Vitamin D And Omega-3 Trial (VITAL) trial [165], and the Vitamin D3–Omega-3-Home Exercise-Healthy Ageing and Longevity (DO-HEALTH) trial [166].

The ViDA included 5108 persons from New Zealand, aged from 50 to 84 years, who were randomized to receive a bolus dose of 100,000 IU cholecalciferol or a placebo monthly. The median follow-up duration was 3.3 yr. The primary endpoint was a composite of cardiovascular disease incidence or death, and it occurred in 11.8% of the intervention group and 11.5% of the placebo group, yielding an adjusted hazard ratio of 1.02 (95% confidence interval (CI) = 0.87–1.20). Similar results were found in relation to baseline vitamin D status and for each of the secondary endpoints.

The VITAL trial recruited 25,871 patients from the USA, with an age of at least 50 yr for men and 55 for women. A factorial 2 × 2 design randomized participants to a daily dose of 2000 IU cholecalciferol and a daily dose of 1 g omega-3 fatty acid, both placebo-controlled. The median follow-up duration was 5.3 yr. The primary endpoints were invasive cancer of any type and major adverse cardiovascular events, and a composite of MI, stroke or death from cardiovascular causes. Results confirmed no significant reduction in either major adverse cardiovascular events or invasive cancer incidence.

The DO-HEALTH trial recruited 2157 adults, with an age of a least 70 years. A factorial 2 × 2 × 2 design was adopted to test cholecalciferol supplement, omega-3 supplement and a strength-exercise program. Supplementation of cholecalciferol was of 2000 IU daily. At a median follow-up of 2.99 yr, the two primary endpoints related to cardiovascular diseases, systolic and diastolic blood pressure, did not achieve statistical significance in relation to the cholecalciferol supplement compared to a placebo.

Altogether, the three large, randomized trials confirmed the absence of clinical benefit in terms of cardiovascular outcomes, despite differences in supplement dosage and follow-up duration. These data were also supported by meta-analyses, indicating the safety but null effect of vitamin D [167,168].

**Table 1:** The main international recommendation thresholds of circulating vitamin D and daily intake levels.

	Target of Circulating 25(OH)-D (nmol/L)	25(OH)-D Insufficiency (nmol/L)	25(OH)-D Deficiency (nmol/L)	Potentially Harmful Levels of Circulating 25(OH)-D (nmol/L)	Recommended Daily Intake of Vitamin D Equivalent (IU/Day)	Upper Tolerable Intake Levels of Vitamin D Equivalent (IU/Day)
Endocrine Society [169]						
Males and females (9–18 years)	75	51–74	≤50	250	600–1000	4000
Males and females (>18 years)	75	51–74	≤50	250	1500–2000	10,000
IOM [170]						
Males and females (9–70 years)	50	30–49	<30	125	600	4000
Males and females (>70 years)	50	30–49	<30	125	800	4000
EFSA 2023 [24]						
Males and females (>18 years)	50	<49		250	600	4000 (10,000) *
Mayo Clinic Recommendation [171]						
Males and females (>18 years)	62.5	25–62.4	<25	200	800–2000	10,000 IU

Converter: 1 µg/L = 1 ng/mL = 2.5 nmol/L. 1 µg = 40 IU. * 10,000 IU is the threshold over which vitamin D toxicity was reported. EFSA suggests considering a 2.5 factor of safety, thus 4000 IU. 25(OH)-D = 25(OH) vitamin D; EFSA = European Food Safety Authority; IOM = Institute of Medicine; IU = international unit.

## 12. Conclusions and Future Perspectives

The numerous differences between the studies and trials conducted so far, in terms of patients’ risk profiles, baseline vitamin D status, supplementation doses, and primary endpoint selection, limit the ability to reach definitive findings and formulate recommendations. The results of pre-clinical studies and the observed significant independent relationships are strong and often concur in favor of a protective role of vitamin D in the cardiovascular field. The discrepancies within interventional clinical studies, both randomized and non-randomized, lead to the hypothesis that further clarification is required in relation to vitamin D metabolism, including genetics investigations. These investigations will be crucial in designing and conducting optimal randomized clinical trials for the appropriate population.
